# Molecular-phylogenetic investigation of trichomonads in dogs and cats reveals a novel *Tritrichomonas* species

**DOI:** 10.1186/s13071-024-06343-0

**Published:** 2024-06-26

**Authors:** Barbara Tuska-Szalay, Julia Gilbert, Nóra Takács, Sándor A. Boldogh, József Fáy, Ágnes Sterczer, Roland Psáder, Jenő Kontschán, Ádám Izsó, Sándor Hornok

**Affiliations:** 1 Department of Parasitology and Zoology, University of Veterinary Medicine, Budapest, Hungary; 2 HUN-REN-UVMB Climate Change, New Blood-Sucking Parasites and Vector-Borne Pathogens Research Group, Budapest, Hungary; 3 Department of Nature Conservation, Aggtelek National Park Directorate, Jósvafő, Hungary; 4 Petcity Animal Clinic, Kecskemét, Hungary; 5 Department of Internal Medicine, University of Veterinary Medicine, Budapest, Hungary; 6 Plant Protection Institute, Centre for Agricultural Research, HUN-REN, Budapest, Hungary; 7 Department of Plant Sciences, Albert Kázmér Faculty of Mosonmagyaróvár, Széchenyi István University, Mosonmagyaróvár, Hungary; 8 Department of Park Rangers, Aggtelek National Park, Jósvafő, Hungary

**Keywords:** *Tritrichomonas foetus*, *Pentatrichomonas hominis*, Rodent, *18S rRNA* gene, ITS

## Abstract

**Background:**

Trichomonosis is a common infection in small animals, mostly manifesting in gastrointestinal symptoms such as diarrhea. Although oral trichomonads are also known, the species found colonizing the large intestine are more frequently detected protozoa.

**Methods:**

In the present study, four wildcats, 94 domestic cats, and 25 dogs, originating from 18 different locations in Hungary, were investigated for the presence of oral and large intestinal trichomonads based on the *18S rRNA* gene and ITS2.

**Results:**

All oral swabs were negative by polymerase chain reaction (PCR). However, *Tritrichomonas foetus* was detected in a high proportion among tested domestic cats (13.8%) and dogs (16%), and *Pentatrichomonas hominis* only in two domestic cats. In addition, a novel *Tritrichomonas* genotype was identified in one cat, probably representing a new species that was shown to be phylogenetically most closely related to *Tritrichomonas casperi* described recently from mice. All positive dogs and half of the positive cats showed symptoms, and among cats, the most frequent breed was the Ragdoll.

**Conclusions:**

With molecular methods, this study evaluated the prevalence of oral and intestinal trichomonads in clinical samples of dogs and cats from Hungary, providing the first evidence of *T. foetus* in dogs of this region. In contrast to literature data, *P. hominis* was more prevalent in cats than in dogs. Finally, a hitherto unknown large intestinal *Tritrichomonas* species (closely related to *T. casperi*) was shown to be present in a cat, raising two possibilities. First, this novel genotype might have been a rodent-associated pseudoparasite in the relevant cat. Otherwise, the cat was actually infected, thus suggesting the role of a predator–prey link in the evolution of this trichomonad.

**Graphical Abstract:**

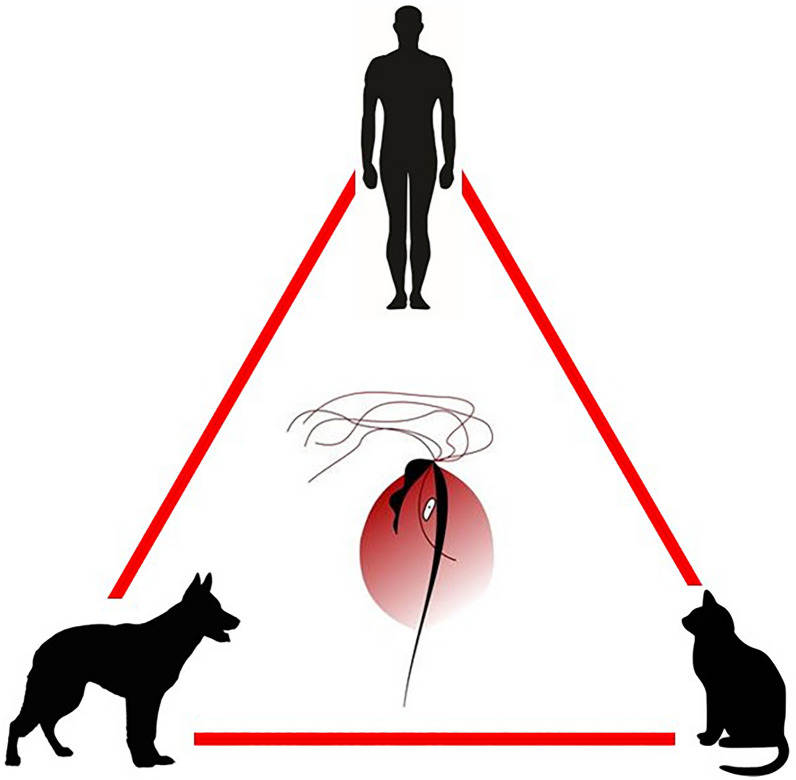

**Supplementary Information:**

The online version contains supplementary material available at 10.1186/s13071-024-06343-0.

## Background

Trichomonads *sensu lato* (Parabasalia: Trichomonadida, Tritrichomonadida) are anaerobic protozoan parasites that live on the mucosal surface of the gastrointestinal tract and reproductive system of both animals and humans. Their multiplication takes place by longitudinal binary division, and the transmission is direct between the hosts. They are highly active flagellates and have only a trophozoite form [1, 2]. However, in an unfavorable condition, they are able to form pseudocysts [3]. Although they are thought to be vulnerable, some of them proved to be more resistant to environmental conditions, since they could survive for 7 days in moist feces at room temperature (23–24 °C) [4].

In dogs and cats, trichomonads might be found in the oral cavity (e.g., *Trichomonas canistomae* in dogs, *Trichomonas felistomae* in cats, and *Trichomonas tenax* in both) [5–7]. In addition, two trichomonads are known to colonize the large intestine of these hosts. One is *Tritrichomonas foetus*, which is able to cause chronic and recurrent diarrhea containing mucus and/or fresh blood in both dogs and cats [1, 2, 8]. The other protozoan is *Pentatrichomonas hominis*, which is considered commensal and opportunistic, and thus its clinical importance has been contested. However, its presence has already been described in dogs and cats in connection with diarrheal symptoms [9, 10]. Based on microscopical examination, these species are difficult to distinguish morphologically from each other, and also from *Giardia duodenalis* which often occurs in co-infection [1, 2, 10].

Several diagnostic methods are available such as direct examination of fresh feces, fecal culture, and polymerase chain reaction (PCR), of which the latter is the most commonly used and most sensitive procedure. Furthermore, there are various approaches in sample collection, including freshly voided stool, manual collection using a fecal loop, or colon flush technique. It is worth noting that the excretion of trophozoites might be intermittent and can be influenced by previous antibiotic therapy [1, 2]. Ronidazole is currently the only effective drug for the treatment of *T. foetus* infection in a dose of 30 mg/kg once daily for a period of 14 days in cats [1, 11]. However, both dogs and cats may be affected by neurotoxic side effects such as lethargy, ataxia, and seizures [12]. The treatment of *P. hominis* is still in question since the infection has been successfully treated with metronidazole in puppies [13], but in kittens, this has not been shown to be effective [14].

Concerning the occurrence of *T. foetus*, which is distributed worldwide and is the most common trichomonad in cats [1], it has already been reported in several countries in Europe using direct examination, fecal culture, or PCR [2]. Considering the European data obtained by PCR, the prevalence of *T. foetus* infection in cats with chronic diarrhea was the highest (38.7%) in Spain [15], followed by 24.4% in Switzerland [16]. In addition, other Western European studies, where not only symptomatic cats were examined, reported *T. foetus* with 15.7% and 5.2% prevalence rates in Germany and Italy, respectively [17, 18]. Within Central and Eastern Europe, the highest prevalence (20.51%) was reported in Poland [19]. However, among neighboring countries of Hungary, the occurrence of *T. foetus* in cats was only reported in Austria with a prevalence of 2.9%, with *P. hominis* also being detected in the study [20].

In comparison with cats, *T. foetus* occurs sporadically in dogs [8, 9, 21], and in Europe this was reported only in Italy [22, 23]. By contrast, *P. hominis* might be more common in dogs than cats, as indicated by its high prevalence of 31.4% in China [9]. Interestingly, relevant data in Europe are scarce, since *P. hominis* was reported only in breeding kennel dogs in France, with 12.1% prevalence, and in a case in Slovenia [24, 25]. In addition, *P. hominis* in cats was detected by PCR only in the United States, Japan, Thailand, Austria, and the Czech Republic [14, 20, 26–29]. Among oral trichomonads in pet animals, apart from *T. canistomae* and *T. felistomae* [6, 7], the zoonotic *T. tenax* has also been detected [5, 30, 31]. Furthermore, a new trichomonad species, *Trichomonas brixi*, has recently been reported in dogs and cats in Czechia [30]. Overall, few data are available on the prevalence of trichomonads in dogs and cats in Central and Eastern Europe. Therefore, the aim of the present study was to determine the presence and prevalence of trichomonads infecting cats and dogs in Hungary.

## Methods

### Sample collection

**Fig. 1:**
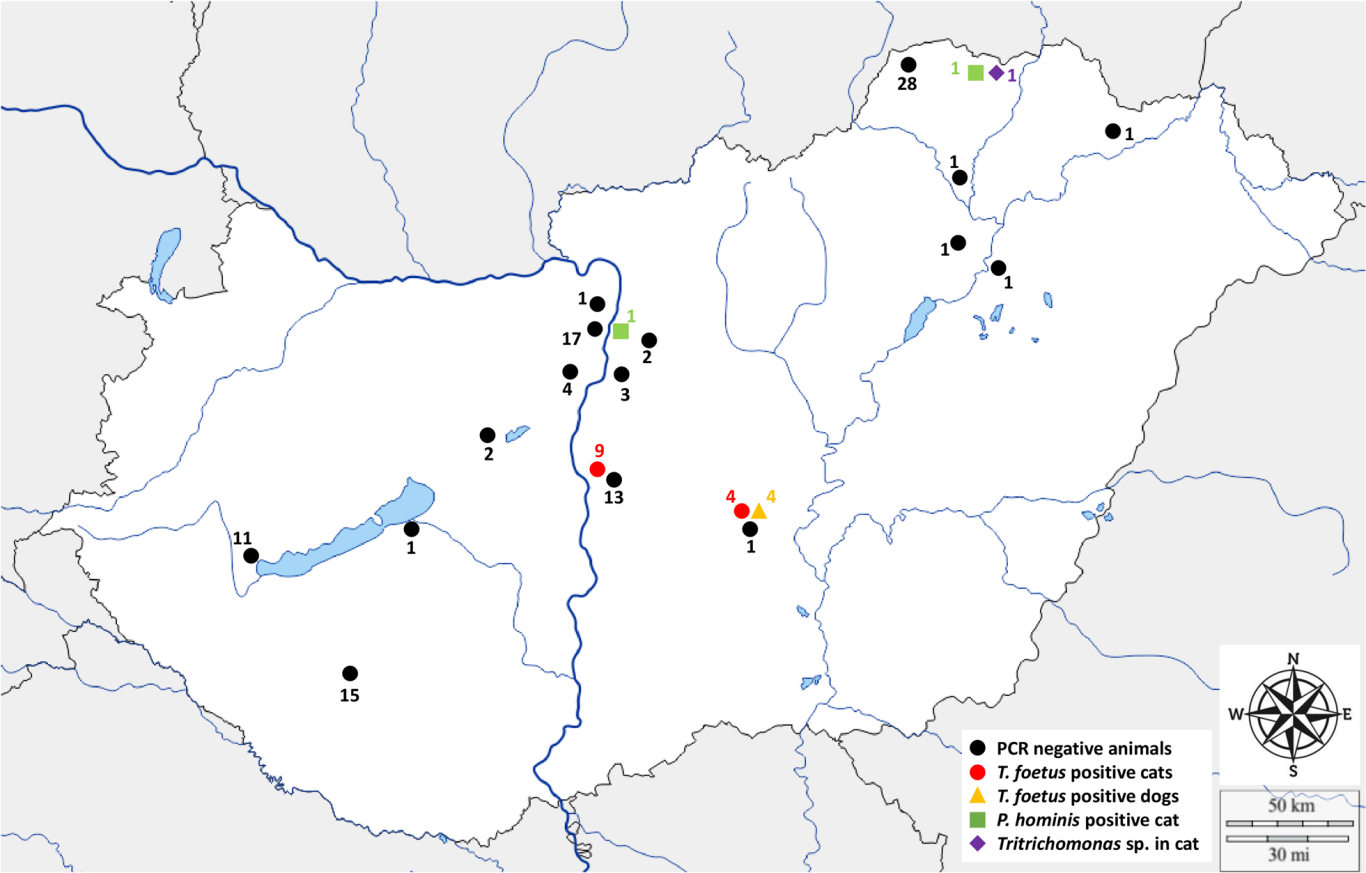
Geographical distribution of samples used in this study. The number next to a mark indicates the number of animals tested at that location

From June 2021 to September 2023, 208 samples were collected from 25 dogs (*Canis lupus familiaris*), 94 domestic cats (*Felis catus*), and four wildcats (*Felis silvestris*) in Hungary. Domestic cat and dog samples originated from 18 locations, including the south-central part of the country (*n* = 31), the capital city Budapest and its surroundings (*n* = 28), Lake Balaton and the surroundings (*n* = 29), and Aggtelek National Park (*n* = 30) (Fig. 1). Wildcats were sampled at the latter location. Sampling of cats in and around the Aggtelek National Park was carried out as part of a targeted sampling campaign for nature conservation purposes.

Five collection methods were applied during the study: fecal swabbing (114), voided feces (45), InPouch® TF-Feline test from Biomed Diagnostics [DCN Dx, Carlsbad, CA, USA (9)], oral swabbing (35), and post-mortem sampling of the intestinal wall of the colon (5), as it is shown in Supplementary Table 1. For the evaluation of sensitivity in detecting intestinal trichomonads, different sampling methods were used simultaneously on a limited number of animals. Of the 114 animals which were sampled with fecal swabs, voided feces (*n* = 40) or samples for InPouch test (*n* = 5) were also collected. Only one cat was sampled with all three methods. During fecal swabbing, a cotton swab was introduced 3–4 cm deep into the rectum of the animal and gently rotated at least three times, connecting it with the rectal wall. A similar procedure was performed for oral swabbing. In addition, intestinal tissue samples were taken from the carcasses of four wildcats and one domestic cat. Swab samples and tissue samples were then placed in sterile Sarstedt tubes. During the collection of fresh feces, it was a prerequisite that the samples should be at least 1 g, free from litter or other contaminants. These freshly voided feces were obtained immediately after excretion and placed in sterile fecal collection containers. The tubes and fecal containers were stored in a freezer (−20 °C) until evaluation. Lastly, the test with InPouch^®^ TF-Feline was performed according to the manufacturer’s instructions.

During the investigation a total of seven different cat breeds were sampled: Ragdoll (41), Devon Rex (1), Maine coon (2), European shorthair (47), Persian- Himalayan (1), British shorthair (1), and Persian (1). Regarding dogs, a higher ratio of mixed breeds were included in the study.

### Data recording

Most of the samples were accompanied by a sample inquiry, to provide information on the location, date of birth, breed, sex, sampling method, collection time, and symptoms (but not treatments). A map of Hungary was created to illustrate the geographical locations of the sampled cats and dogs (Fig. 1).

### DNA extraction, PCR, and sequencing

DNA was extracted using the QIAamp^®^ Fast DNA Stool Mini Kit (QIAGEN, Hilden, Germany) according to the manufacturer’s instructions, with some modifications—i.e., prior to adding Buffer AL, the solution was incubated at 56 °C for 60 min, and then the Buffer AW1 was used twice during the washing procedure. During DNA extractions, each set of samples included an extraction control to monitor cross-contamination. DNA extracts were stored at −20 °C until molecular analysis by conventional PCR.

All samples were screened for the short fragment of the 18S ribosomal RNA (rRNA) gene, then only the positive samples were examined further with PCRs for the long fragment of the *18S rRNA* gene and internal transcribed spacer 2 (ITS2). For each PCR reaction, a total volume of 25 µl was used, which consisted of 5 µl of extracted DNA and 20 µl of reaction mixture. The latter contained 0.2 µl HotStar Taq Plus DNA Polymerase (Qiagen, Hilden, Germany), 0.5 µl dNTP mix (10 mM), 1 µM of each primer, 2.5 µl of 10× CoralLoad PCR buffer (15 mM MgCl2 included) and 15.8 µl PCR-grade water. Further details of the PCRs are summarized in Table 1 [32–34]. In all PCRs, sequence-verified positive controls were included, as well as non-template reaction mixture as negative control. PCR products were electrophoresed in 1.5% agarose gel (100 V, 55–60 min), stained with ethidium bromide, and visualized under ultraviolet (UV) light.

**Table 1:** Primers and details for conventional PCR methods used in this study

Target group	Target gene	Primer name	Primer sequence (5′–3′)	Amplicon length (base pairs)	Thermal cycling profile	References
Trichomonadidae screening assay for short fragment	*18S rRNA*	1055F 16SR1	GGT GGT GCA TGG CCG TCA CCT ACC GTT ACC TTG	~ 500	95 °C for 5 min; 40 × (95 °C for 45 s; 50 °C for 45 s; 72 °C for 1.5 min); 72 °C for 10 min	[32]
Trichomonadidae semi-nested PCR assay for long fragments	*18S rRNA* outer	16SL 16SR1	TAC TTG GTT GAT CCT GCC TCA CCT ACC GTT ACC TTG	~ 1550	95 °C for 5 min; 45 × (95 °C for 45 s; 48 °C for 45 s; 72 °C for 1.5 min); 72 °C for 10 min	[33]
	*18S rRNA* nested	16SL 1385R	TAC TTG GTT GAT CCT GCC GAT CCT AAC ATT GTA GC	~ 1450	95 °C for 5 min; 45 × (95 °C for 45 s; 42 °C for 45 s; 72 °C for 1.5 min); 72 °C for 10 min;	
Trichomonadidae	ITS2	TFR1 TFR2	TGC TTC AGT TCA GCG GGT CTT CC CGG TAG GTG AAC CTG CCG TTG G	~ 330–380	95 °C for 5 min; 40 × (95 °C for 30 s; 65 °C for 30 s; 72 °C for 50 s); 72 °C for 5 min	[34]

Purification of selected PCR products and sequencing in one direction were performed by Eurofins Biomi Ltd. (Gödöllő, Hungary). Quality control and trimming of sequences were performed using the BioEdit program (Informer Technologies, Inc.). Obtained sequences were compared to GenBank sequences using the Basic Local Alignment Search Tool nucleotide (BLASTN) program (https://blast.ncbi.nlm.nih.gov). Unique sequences obtained in this study were submitted to GenBank (Accession Numbers: PP227421-PP227425 for the *18S rRNA* gene, PP239334-PP239337 for ITS2). Further details are shown in Supplementary Table 2.

### Phylogenetic analysis

All sequences retrieved from GenBank for the phylogenetic analyses had 99–100% coverage with sequences from this study and were trimmed to the same length. Thus, sequences included in the phylogenetic analysis of the *18S rRNA* gene (*n* = 36) represented six orders of Trichomonadea. However, the availability of sequences in GenBank covering the same length of ITS2 as amplified in this study, limited the number of sequences to 19 that could be used in the relevant phylogenetic analysis. The sequence datasets were resampled 1000 times to generate bootstrap values. The method and the model that were used to infer the evolutionary history are indicated in figure captions. The percentage of trees in which the associated taxa clustered together is shown next to the branches. The trees were drawn to scale, with branch lengths measured in the number of substitutions per site. All positions containing gaps and missing data were eliminated. Evolutionary analyses were conducted in MEGA7.

### Data analysis

First, a standard descriptive statistical analysis was used to review the acquired data including prevalence, mean, and median. Comparisons between different factors (sex, age, breed, symptom) were performed with the Fisher exact test (https://www.langsrud.com/fisher.htm).

## Results

### Molecular identification and phylogenetic analyses of trichomonads

Altogether 123 animals were PCR-tested, among which 20 were positive for trichomonads (Table 2). No significant difference was found in the rate of PCR positivity according to sampling methods. Thirteen (13.8%) of domestic cats were positive for *T. foetus* and two (2.1%) for *P. hominis*. In addition, one feline sample (1.1%) contained the DNA of a different *Tritrichomonas* species which in the sequenced, 337-bp-long part of its *18S rRNA* gene (PP227424) was genetically most closely related to *Tritrichomonas casperi* (ON927245) isolated from mice (*Mus musculus*), showing 96.44% identity to the latter. Regarding dogs, four out of 25 (16%) proved to be *T. foetus*-positive. Wildcats did not harbor any trichomonads.

**Table 2:** Data for the 20 animals found positive for Trichomonadidae by PCR

Site	Animal	Breed	Age^a^	Sex^a^	Symptoms	Sampling	PCR result
Kunszentmiklós	Cat	Ragdoll	2 y	F	No	Feces + swab + InPouch	*T. foetus*
Kunszentmiklós	Cat	Ragdoll	1.5 y	F	No	Swab	*T. foetus*
Kunszentmiklós	Cat	Ragdoll	2 y	F	No	Swab	*T. foetus*
Kunszentmiklós	Cat	Ragdoll	3 y	M	No	Swab	*T. foetus*
Kunszentmiklós	Cat	Ragdoll	1.5 y	F	Diarrhea	Swab	*T. foetus*
Kunszentmiklós	Cat	Ragdoll	4 y	F	No	Swab	*T. foetus*
Kunszentmiklós	Cat	Ragdoll	1 y	M	No	Swab	*T. foetus*
Kunszentmiklós	Cat	Ragdoll	2 m	F	Diarrhea	InPouch	*T. foetus*
Kunszentmiklós	Cat	Ragdoll	4 m	M	Diarrhea	InPouch	*T. foetus*
Budapest	Cat	Persian-Himalayan	7 m	F	Strong diarrhea	Feces + swab	*P. hominis*
Komjáti	Cat	European shorthair	1 y	F	Unknown	Swab	*Tritrichomonas* sp.
Komjáti	Cat	European shorthair	6 m	F	Unknown	Swab	*P. hominis*
Kecskemét	Cat	Ragdoll	3 m	M	Diarrhea	Swab	*T. foetus*
Kecskemét	Cat	Ragdoll	3 m	F	Diarrhea	Swab	*T. foetus*
Kecskemét	Cat	Ragdoll	3 m	F	Diarrhea	Swab	*T. foetus*
Kecskemét	Cat	Ragdoll	3 m	F	Diarrhea	Swab	*T. foetus*
Kecskemét	Dog	Maltese dog	3 m	M	Diarrhea	Swab	*T. foetus*
Kecskemét	Dog	Unknown	Unknown	Unknown	Diarrhea	Swab	*T. foetus*
Kecskemét	Dog	Unknown	Unknown	Unknown	Diarrhea	Swab	*T. foetus*
Kecskemét	Dog	Unknown	Unknown	Unknown	Diarrhea	Swab	*T. foetus*

^*a*^*m* months, *y* years, *M* male, *F* female

All six feline isolates of *T. foetus*, from which a longer part of the *18S rRNA* gene was successfully amplified, had 100% sequence identity to each other (PP227421) and to sequences of *T. foetus* deposited in GenBank from cats (AF466749) and cattle (AY055799), as well as that of *Tritrichomonas suis* from pigs (MK801504). The same can be said for the 17 *T. foetus*-positive samples (PP227422, PP227423), in which the short part of *18S rRNA *was examined. Based on the examination of ITS2, the sequence of *T. foetus* from this study (PP239334), 100% sequence identity was shown to *T. foetus* sequences of cats from China (OP866181 and OP856640) and the USA (AF466749).

Considering the short *18S rRNA* sequence of the two *P. hominis*-positive cat samples (PP227425) it showed 100% identity to a *P. hominis* isolate from a cat (KC594038) and 99.3% identity to *P. hominis* from a dog (AY758392). The longer part of the *18S rRNA* gene could not be amplified from these samples. Regarding the corresponding ITS2 sequence (PP239337), it was 99.7% identical to *P. hominis* of a cat from Czechia (KC594038) and of a dog from the USA (AY758392). In addition, the ITS2 also showed 99.7% sequence identity to *P. hominis* of a human sample from Thailand (AF156964). All data are summarized in Supplementary Table 2.

**Fig. 2:**
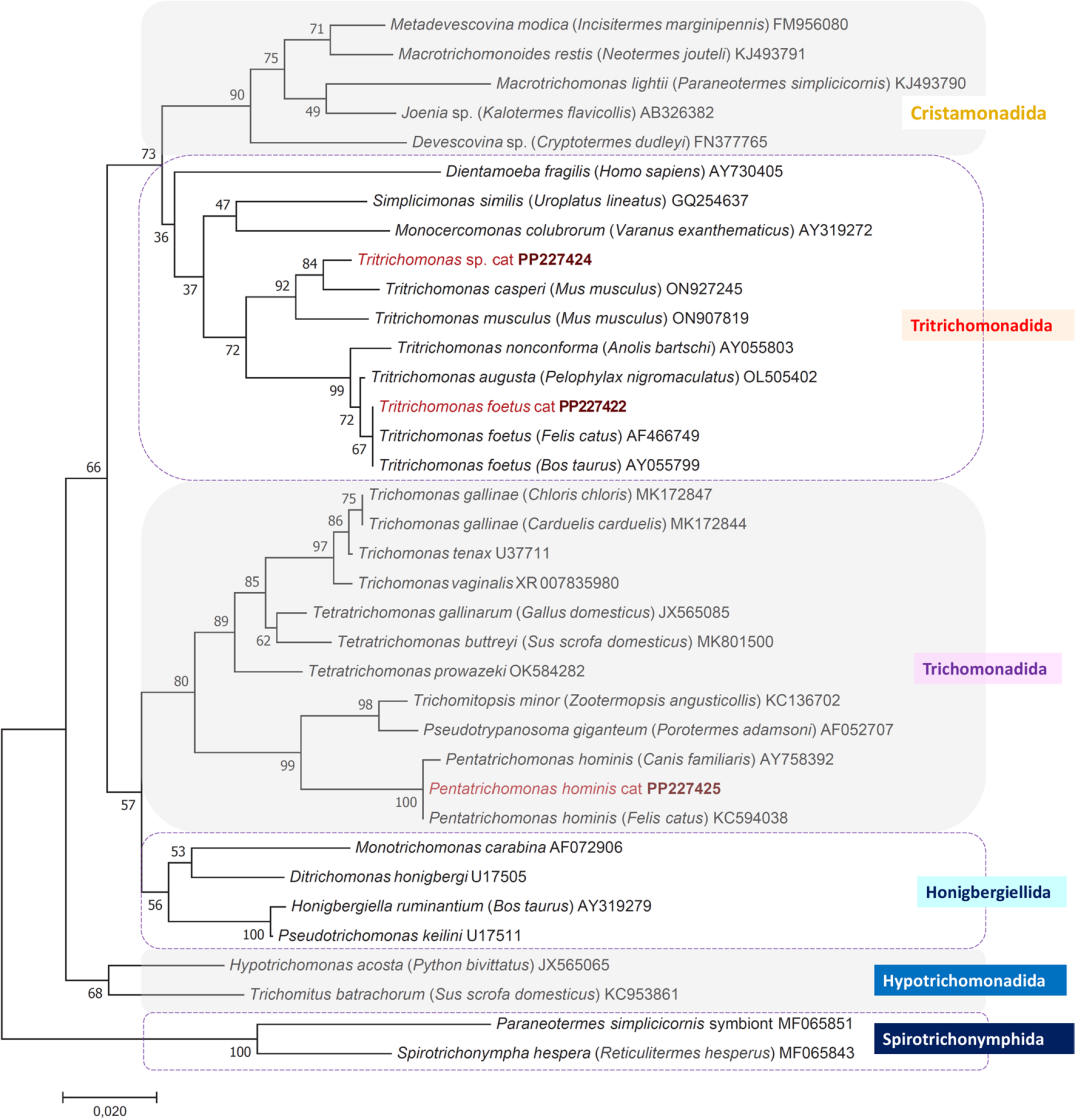
Phylogenetic tree of Trichomonadea based on the *18S rRNA* gene, made with the neighbor-joining method and p-distance model. In each row, after the species or genus name, the isolation source of trichomonads and the GenBank accession number are shown. Sequences obtained in this study are in red, with bold accession numbers. The analysis involved 36 nucleotide sequences. The final length of the alignment was 281 bp. Two species of Spirotrichonymphida were used as outgroup. The scale bar indicates the number of substitutions per site

Based on the results of the short *18S rRNA* gene and ITS2 phylogenetic analyses, all *T. foetus* sequences from cats and cattle clustered together, including those from this study (PP227422 and PP239334, respectively), with moderate to high support (Fig. 2, Supplementary Figure 1). Similarly, based on both genetic markers, *P. hominis* sequences from this study (PP227425 and PP239337, respectively) belonged to the phylogenetic group of *P. hominis* isolates from cats, dogs and human with high (100%) support (Fig. 2, Supplementary Figure 1). In line with the molecular comparisons described above, phylogenetic analyses of the *18S rRNA *gene and ITS2 sequences of the novel *Tritrichomonas* genotype obtained from a cat in northeastern Hungary (PP227424: 337-bp-long, and PP239336: 342-bp-long, respectively) showed that it is a sister species of *T. casperi* from mice (*Mus musculus*) (Fig. 2, Supplementary Figure 1).

### Geographical distribution of positive samples

All 13 *T. foetus*-positive domestic cats and four dogs were from the south-central region of Hungary. One of the *P. hominis*-positive cats was from Budapest, and the other was from Aggtelek National Park, similar to the *Tritrichomonas* species-infected cat (Table 2, Fig. 1).

### Analysis of host data and morbidity

The sex of 90 cats (95.7% of all) and 21 dogs (84% of all) in the study was known. Based on their data, the sex ratio was 65.6% (*n* = 59) females and 34.4% (*n* = 31) males among cats, and 42.9% (*n* = 9) females and 57.1% (*n* = 12) males among dogs. Age was reported for 68 (72.3%) of the domestic cats and 22 (88%) of the dogs in the investigation. The mean age of the cats was 6.47 years (median 6 years), ranging from kittens to 14 years of age. The mean age of dogs was 6.3 years (median 6 years). In total, 17% (*n* = 16) of cats and 32% (*n* = 8) of dogs showed symptoms of gastrointestinal disorder.

Data for PCR-positive cats and dogs are summarized in Table 2. Among the *T. foetus*-positive cats (*n* = 13), there were more females (69.2%) than males (30.8%), while both *P. hominis*-positive cats were females. Based on this, there was no significant correlation between PCR positivity and sex. On the other hand, the rate of PCR positivity was significantly (*P* = 0.0011) higher among Ragdoll cats (13 of 41: 31.7%) than among European shorthair cats (2 of 47: 4.3%). The mean ages of *T. foetus*- and *P. hominis*-infected cats were 15.2 (median 12), and 6.5 (median 6.5) months, respectively. This implies that significantly (*P* = 0.0273) more cats were PCR-positive below 1 year of age (9 of 21: 42.9%), than among older cats (7 of 47: 14.6%). Among *T. foetus*-positive dogs, the age and sex were provided for only one dog (3-month-old male).

Out of 16 PCR-positive cats, eight (50%) showed clinical symptoms, mainly diarrhea (Table 2). Thus, PCR-positive cats showed gastrointestinal symptoms significantly (*P* = 0.0011) more frequently than negative cats (9 of 78: 11.5%). At the same time, all the positive dogs showed relevant clinical signs.

## Discussion

Trichomonosis is a widespread parasitic infection in cats, and its most frequent causative agent is *T. foetus*, as reported in several countries [10]. However, relevant data were not available from Hungary, and some countries of its geographical region, justifying the need for a survey described in the present study. In addition, molecular phylogenetic data on other trichomonads of dogs and cats are limited even in a worldwide context.

In the present study, four wildcats from Aggtelek National Park were screened for oral and intestinal trichomonads. Although trichomonad DNA was not found, the opportunity to be infected was given, since in that region outdoor domestic cats and wildcats have been proven to share their living space with each other. This is also supported by recent reports of *Hepatozoon felis* in wildcats and domestic cats in the same region. [35, 36].

On the other hand, domestic cats sampled in this study showed 13.8% prevalence of *T. foetus*. Although in Western Europe the occurrence of *T. foetus* seems to be more common, its presence in Central and Eastern Europe cannot be neglected. Based on the studies using PCR, the highest prevalence (38.7%) was reported in Spain, among 93 densely housed cats with chronic diarrhea [15]. In Switzerland, 10 out of 45 cats with diarrhea proved to be positive for *T. foetus* [16]. Furthermore, in Germany, 15.7% of 230 purebred cats were PCR-positive but only 61% of them showed diarrheic symptoms [17]. In Italy, 267 cats kept in different environments were screened and 14 of them (5.2%) were clinically *Tritrichomonas*-infected [18]. Among neighboring countries, similar studies were conducted. However, *T. foetus*-positive cats were found only in Austria, with 2.9% prevalence [20, 29]. In addition, in the northern part of Central Europe (Poland), one clinical case was reported [37].

In dogs, the occurrence of *T. foetus* is not as common as in cats. This is supported by data from different continents, i.e., from East China and the United States, where *T. foetus* was reported with only 0.6% and 2.6% prevalence in dogs, respectively [9, 21]. To the best of our knowledge, this is the first report of *T. foetus* in dogs in Europe north of the Mediterranean Basin, since previously this has only been reported in Italy: once in 2018 when one out of 100 shelter dogs proved to be infected [22], then in 2020 when *T. foetus* was found in an atypical location, i.e., in a subcutaneous mass of a dog [23]. Among the 25 dogs in the present study, four were positive for *T. foetus* and all had diarrhea. This observation may contradict the statement that *P. hominis* is more frequent than *T. foetus* in dogs with diarrhea [21]. Since symptomatic trichomonosis appears between 7 weeks and 6 months of age [21], this corresponds well to that of the PCR-positive dog for which the age was known (3 months). In contrast, in another study, two adult dogs were positive for *T. foetus* among 38 diarrheic dogs, with one of them being co-infected with *P. hominis* [21]. Similarly, in East China, two adult (> 12-month-old) dogs out of 315 proved to be positive for *T. foetus*, and one of them had diarrhea [9].

While *T. foetus* is a protozoon with pathogenic potential, until recently *P. hominis* has been considered a non-pathogenic opportunistic parasite in different mammalian hosts including dogs, cats, and humans [38]. Hence all dogs and cats infected with *P. hominis* have the potential for zoonotic transmission [27]. Some recently published studies have reported an association between *P. hominis* infection and the occurrence of diarrhea [10, 13, 39], which can also be supported by our results, since one of the two *P. hominis* positive cats had strong diarrhea. Whether *P. hominis* can cause large bowel diarrhea by itself or only in co-infection with other parasites is still unknown [14, 26]. It is noteworthy that this protozoon is frequently misidentified as *T. foetus*; therefore, its veterinary medical significance is probably underestimated [26]. In the present study, *P. hominis* was identified in 2.1% of the cats, one of which (a 7-month-old Persian-Himalayan cat) had diarrhea. This is in line with an American study that also revealed the presence of *P. hominis* in diarrheic young purebred cats [14]. *Pentatrichomonas hominis* is known to be a less frequently observed protozoon in cats than *T. foetus* [20, 26, 27]. Thus, not surprisingly, in Europe only a few reports have hitherto provided data on *P. hominis* in cats [20, 29]. Therefore, this is the third study in Europe showing a potential pathogenic role of *P. hominis* in cats. In addition, based on the ITS2 phylogenetic tree, the feline *P. hominis* isolate clustered together with *P. hominis* from a human sampled in Thailand. This supports the theory that *P. hominis* is a zoonotic parasite, although its zoonotic transmission still has to be proved [27, 38].

In this study, the swab sample of a single female cat without any symptoms contained the DNA of a novel *Tritrichomonas* genotype or species which showed the highest (96.44%) identity to *T. casperi* and clustered as its sister species on both phylogenetic trees. *Tritrichomonas casperi* was reported to colonize the caecum of a laboratory mouse (*Mus musculus*) [40]. The relevant sample in this study was obtained from the rectum via mucosal swabbing; therefore, it was probably associated with epicellular parasitism and not with a digested prey item as a pseudoparasite. However, the latter cannot be completely excluded, as this finding can still be associated with mice eaten by the relevant cat, thus originating from a rodent-associated trichomonad. Nevertheless, since felines are not known as natural hosts of *T. casperi* described from mice, data should be interpreted with caution, especially considering the short size (337 bp) of the *18S rRNA* sequence used in our phylogenetic analysis. If confirmed, the detection of DNA from a *T. casperi*-related species from cats could indicate a possible role of a predator–prey link in the evolution of this feline trichomonad, similar to what is known of avian trichomonosis [41]. Further studies are needed to confirm the identity of this *Tritrichomonas* species and its phylogenetic relationship with *T. casperi*.

Although *T. foetus* usually infects young (< 12 months) animals [1, 9, 37], this could not be precisely confirmed in our study, as there were PCR-positive cats of different ages. The mean age was more than 12 months among positive cats, which can be explained by the fact that some positive Ragdoll cats were from the same cattery, including older cats which were certainly asymptomatic carriers. Concerning the breed of *T. foetus*-infected cats, only the Ragdoll and European shorthair breeds yielded positive results. However, no conclusion can be drawn from this, since more than 93% of the cats in this study were from these two breeds. In line with this, cats from catteries or shelters are at increased risk of becoming infected [1]; for example, in the UK and Norway, *T. foetus* has also been reported in other breeds such as Siamese, Bengal, and Burmese along with the Ragdoll [42, 43]. In addition, in Germany, Norwegian Forest cats were the most commonly infected among other breeds [17].

In this survey, no significant association was observed between trichomonad infection and the sex of animals, confirming the results of the above studies, because in the context of *T. foetus* infection, the sex was not reported as a predisposing factor. However, a significant association was found here between the presence of *T. foetus* DNA and gastrointestinal symptoms at the time of sampling, or with cats having a history of signs of enteritis. This is in line with previous studies, where symptoms played a key role in the sampling of *T. foetus*-positive cats [17, 44]. This is particularly relevant at a young age, because older infected cats may be asymptomatic carriers with a long history of diarrhea in kittenhood [1], as also shown here. In addition, if cats are kept together in breeding facilities, they are more likely to contract *T. foetus* infection, as was found among Ragdoll cats in this study.

## Conclusions

This study evaluated the prevalence of intestinal trichomonads in clinical samples of dogs and cats in Hungary and its region. When interpreting the results, it has to be taken into account that the presence of pathogen DNA in the fecal sample does not necessarily mean that the animal was infected. Furthermore, even the combination of diarrhea and the presence of trichomonad DNA in the feces can be a coincidence, and the clinical signs might be attributed to a number of causes. Here, the presence of *T. foetus* was established for the first time in dogs in Central and Eastern Europe. Furthermore, *T. foetus* and *P. hominis* were also detected with relatively high prevalence in cats. Based on the results, we conclude that the prevalence of *T. foetus* and *P. hominis* appears to be highly variable among the cat population examined, with infections being more common in pedigrees from catteries. Besides *T. foetus* and *P. hominis*, we detected a possible different *Tritrichomonas* species in a cat. This putative species seems to be related to *T. casperi* of mice, but this finding requires confirmation.

### Supplementary Information

Supplementary material 1 Figure S1. Phylogenetic tree of Trichomonadea based on ITS2 sequences, constructed with the maximum likelihood method and the Jukes–Cantor model. In each row, after the species or genus name, the isolation source, the country of origin, and the GenBank accession number are shown. Sequences obtained in this study are in red, with bold accession numbers. The analysis involved 19 nucleotide sequences. The final length of the alignment was 296 bp. No outgroup was used. The scale bar indicates the number of substitutions per site.

Supplementary material 2 Table S1. Location and mode of sample collection according to data of animals involved in this study.

Supplementary material 3 Table S2. Distribution of PCR-positive and sequenced samples along with the GenBank accession numbers.

## Data Availability

The sequences obtained during this study are deposited in GenBank under the following accession numbers: *18S rRNA* gene: PP227421–PP227425, ITS2 gene: PP239334–PP239337. All other relevant data are included in the manuscript and the references or are available upon request by the corresponding author.

## Abbreviation

ITS2: Internal transcribed spacer 2
bp: base pair
