# Supplementary figures and images for: Molecular-phylogenetic investigation of trichomonads in dogs and cats reveals a novel Tritrichomonas species
Source: Parasit Vectors. 2024 Jun 26;17:271. doi: 10.1186/s13071-024-06343-0 (PMC11210186; doi:10.1186/s13071-024-06343-0)

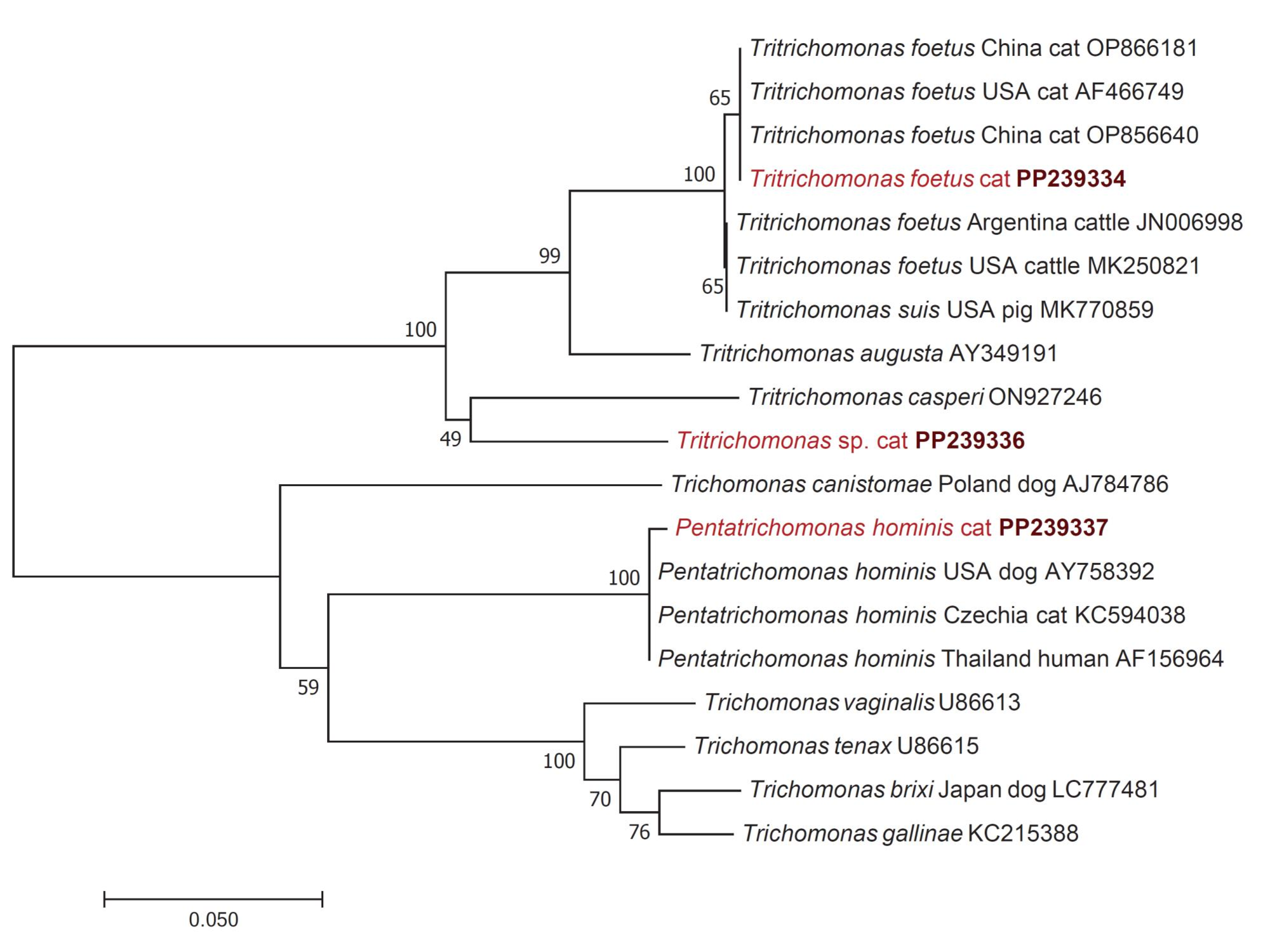

Supplement: Supplementary file 1 — Supplementary material 1 Figure S1. Phylogenetic tree of Trichomonadea based on ITS2 sequences, constructed with the maximum likelihood method and the Jukes–Cantor model. In each row, after the species or genus name, the isolation source, the country of origin, and the GenBank accession number are shown. Sequences obtained in this study are in red, with bold accession numbers. The analysis involved 19 nucleotide sequences. The final length of the alignment was 296 bp. No outgroup was used. The scale bar indicates the number of substitutions per site. [file 13071_2024_6343_MOESM1_ESM.tiff]
