# Supplementary material for: Molecular-phylogenetic investigation of trichomonads in dogs and cats reveals a novel Tritrichomonas species
Source: Parasit Vectors. 2024 Jun 26;17:271. doi: 10.1186/s13071-024-06343-0 (PMC11210186; doi:10.1186/s13071-024-06343-0)
Supplement: Supplementary file 2 — Supplementary material 2 Table S1. Location and mode of sample collection according to data of animals involved in this study. [file 13071_2024_6343_MOESM2_ESM.pdf]

**Supplementary Table 1.** Data of animals sampled in this study.													
**sample**	**location**	**fecal swab**	**feces**	**InPouch**	**oral swab**	**wall of colon**	**species**	**breed**	**<1 year**	**sex**	**GI symptoms**	**PCR**	**sequencing**
Szemi	Kunszentmiklós	-	+	-	-	-	cat	Ragdoll		female	no	negative	
**Parfi**	**Kunszentmiklós**	+	+	+	-	-	**cat**	**Ragdoll**		**female**	**no**	**positive**	**100% *T. foetus***
Geszti Eszti	Kunszentmiklós	+	+	-	-	-	cat	Ragdoll		female	no	negative	
Incike	Kunszentmiklós	-	+	-	-	-	cat	Ragdoll		male	no	negative	
Owen	Kunszentmiklós	-	+	-	-	-	cat	Ragdoll	+	male	no	negative	
Randy	Kunszentmiklós	+	-	-	-	-	cat	Ragdoll		female	no	negative	
**Sugi**	**Kunszentmiklós**	+	-	-	-	-	**cat**	**Ragdoll**		**female**	**no**	**positive**	**100% *T. foetus***
Ari	Kunszentmiklós	+	-	-	-	-	cat	Ragdoll		female	no	negative	
**Mrs Boss**	**Kunszentmiklós**	+	-	-	-	-	**cat**	**Ragdoll**		**female**	**no**	**positive**	**100% *T. foetus***
Mignon	Kunszentmiklós	+	-	+	-	-	cat	Devon rex		female	no	negative	
Arany Mazs	Kunszentmiklós	+	-	-	-	-	cat	Ragdoll		female	no	**negative**	**100% *T. foetus***
**Handy**	**Kunszentmiklós**	+	-	-	-	-	**cat**	**Ragdoll**		**male**	**no**	**positive**	**100% *T. foetus***
**Izabella**	**Kunszentmiklós**	+	-	+	-	-	**cat**	**Ragdoll**		**female**	**yes**	**positive**	**100% *T. foetus***
**Grut**	**Kunszentmiklós**	+	-	-	-	-	**cat**	**Ragdoll**		**female**	**no**	**positive**	**100% *T. foetus***
**Berci**	**Kunszentmiklós**	+	-	-	-	-	**cat**	**Ragdoll**		**male**	**no**	**positive**	**100% *T. foetus***
Boglárka	Kunszentmiklós	+	-	+	-	-	cat	Ragdoll		female	no	negative	
Luker	Kunszentmiklós	+	-	+	-	-	cat	Ragdoll		male	no	negative	
Dolci	Kunszentmiklós	+	-	-	-	-	cat	Ragdoll		female	no	negative	
296716	Budapest	+	+	-	-	-	cat	Ragdoll		male	yes	negative	
Zafír	Budapest	+	-	-	-	-	cat	European shorthair	+	-	yes	negative	
Kissugi	Kunszentmiklós	-	-	+	-	-	cat	Ragdoll	+	female	yes	negative	
**Szép Kissugi**	**Kunszentmiklós**	-	-	+	-	-	**cat**	**Ragdoll**	+	**female**	**yes**	**positive**	**100% *T. foetus***
Tina	Kunszentmiklós	-	-	+	-	-	cat	Ragdoll		female	no	negative	
**Legacy**	**Kunszentmiklós**	-	-	+	-	-	**cat**	**Ragdoll**	+	**male**	**yes**	**positive**	**100% *T. foetus***
**308470**	**Budapest**	+	+	-	-	-	**cat**	**Persian-himalayen**	+	**female**	**yes**	**positive**	**100% *P. hominis***
1	Aggtelek	+	-	-	+	-	cat	European shorthair		male	no	negative	
2	Aggtelek	+	-	-	+	-	cat	European shorthair		female	no	negative	
3	Aggtelek	+	-	-	+	-	cat	European shorthair	+	male	no	negative	
4	Aggtelek	+	-	-	+	-	cat	European shorthair		male	no	negative	
5	Aggtelek	+	-	-	+	-	cat	European shorthair		female	no	negative	
6	Aggtelek	+	-	-	+	-	cat	European shorthair		male	no	negative	
7	Aggtelek	+	-	-	+	-	cat	European shorthair		female	no	negative	
8	Aggtelek	+	-	-	+	-	cat	European shorthair	+	female	no	negative	
9	Aggtelek	+	-	-	+	-	cat	European shorthair	+	male	no	negative	
10	Aggtelek	+	-	-	+	-	cat	European shorthair		female	no	negative	
11	Aggtelek	+	-	-	+	-	cat	European shorthair		male	no	negative	
12	Aggtelek	+	-	-	+	-	cat	European shorthair	+	male	no	negative	
13	Aggtelek	+	-	-	+	-	cat	European shorthair		female	no	negative	
14	Aggtelek	+	-	-	+	-	cat	European shorthair		male	no	negative	
15	Aggtelek	+	-	-	+	-	cat	European shorthair		female	no	negative	
**16**	**Aggtelek**	+	-	-	+	-	**cat**	**European shorthair**	+	**female**	**no**	**positive**	***Tritrichomonas* sp. (96,44% *T. casperi*)**
17	Aggtelek	+	-	-	+	-	cat	European shorthair		female	no	negative	

18	Aggtelek	+	-	-	+	-	cat	European shorthair		female	no	negative	
19	Aggtelek	+	-	-	+	-	cat	European shorthair		male	no	negative	
**20**	**Aggtelek**	**+**	-	-	**+**	-	**cat**	**European shorthair**	+	**female**	**no**	**positive**	**100% *P. hominis***
21	Aggtelek	+	-	-	+	-	cat	European shorthair		male	no	negative	
22	Aggtelek	+	-	-	+	-	cat	European shorthair		female	no	negative	
23	Aggtelek	+	-	-	+	-	cat	European shorthair		-	no	negative	
24	Aggtelek	+	-	-	+	-	cat	European shorthair		female	no	negative	
25	Aggtelek	+	-	-	+	-	cat	European shorthair		female	no	negative	
26	Aggtelek	+	-	-	+	-	cat	European shorthair		female	no	negative	
27	Aggtelek	+	-	-	+	-	cat	European shorthair		male	no	negative	
28	Aggtelek	+	-	-	+	-	cat	European shorthair		male	no	negative	
29	Aggtelek	+	-	-	+	-	cat	European shorthair		female	no	negative	
30	Aggtelek	+	-	-	+	-	cat	European shorthair		female	no	negative	
WC21	Bodrogolaszi	+	-	-	+	+	wildcat	Felis silvestris		-	no	negative	
WC22	Újszentmargita	+	-	-	+	+	wildcat	Felis silvestris		-	no	negative	
WC23	Múcsony	+	-	-	+	+	wildcat	Felis silvestris		-	no	negative	
WC24	-	+	-	-	+	+	wildcat	Felis silvestris		-	no	negative	
WC25	Ónod	+	-	-	+	+	cat	European shorthair		-	no	negative	
308799	Érd	-	+	-	-	-	cat	Ragdoll	+	female	yes	negative	
304950	Budapest	+	+	-	-	-	dog	French bulldog		female	no	negative	
305097	Harkány	+	+	-	-	-	dog	Yorkshire terrier		male	no	negative	
304761	Székesfehérvár	+	+	-	-	-	cat	British shorthair		female	yes	negative	
305722	Budapest	+	+	-	-	-	dog	vizsla		female	yes	negative	
301624	Érd	+	+	-	-	-	dog	Bichon havanais		female	yes	negative	
294346	Budaörs	+	+	-	-	-	dog	Labrador retriever		male	no	negative	
305916	Budapest	+	+	-	-	-	dog	Sheltie		male	no	negative	
305989	Siófok	+	+	-	-	-	dog	Coton de tulier		male	no	negative	
264828	Budapest	+	+	-	-	-	dog	Border collie		male	no	negative	
306200	Mogyoród	+	+	-	-	-	dog	mix		male	no	negative	
306211	Budapest	+	+	-	-	-	dog	mix		female	no	negative	
273987	Budapest	+	+	-	-	-	dog	Golden retriever		female	no	negative	
306320	Budapest	+	+	-	-	-	cat	European shorthair		female	yes	negative	
306416	Ecser	+	+	-	-	-	cat	European shorthair		male	yes	negative	
306459	Budapest	+	+	-	-	-	dog	Bichon havanais	+	female	no	negative	
306480	Budapest	+	+	-	-	-	cat	Persian		male	no	negative	
306472	Budapest	+	+	-	-	-	dog	mix		female	no	negative	
306502	Érd	+	+	-	-	-	dog	mix		female	no	negative	
306584	Budapest	+	+	-	-	-	dog	Cairn terrier		male	no	negative	
306648	Veresegyház	+	+	-	-	-	dog	Labrador retriever		female	no	negative	
306746	Budapest	+	+	-	-	-	cat	European shorthair		male	no	negative	
306914	Csemő	+	+	-	-	-	dog	mix		male	yes	negative	
306924	Szigetszentmiklós	+	+	-	-	-	dog	Mops		male	no	negative	
306906	Budapest	+	+	-	-	-	dog	vizsla		male	no	negative	
307827	Budapest	+	+	-	-	-	cat	European shorthair		male	no	negative	

307951	Székesfehérvár	+	+	-	-	-	cat	Maine coon		male	no	negative	
306511	Budapest	+	-	-	-	-	dog	Border collie		male	no	negative	
1.	Kaposvár	+	+	-	-	-	cat	Ragdoll	+	male	no	negative	
2.	Kaposvár	+	+	-	-	-	cat	Ragdoll		female	no	negative	
3.	Kaposvár	+	+	-	-	-	cat	Ragdoll	+	male	no	negative	
4.	Kaposvár	+	+	-	-	-	cat	Ragdoll		female	no	negative	
5.	Kaposvár	+	+	-	-	-	cat	Ragdoll		female	no	negative	
6.	Kaposvár	+	+	-	-	-	cat	Ragdoll		male	no	negative	
7.	Kaposvár	+	+	-	-	-	cat	Ragdoll		female	no	negative	
8.	Kaposvár	+	-	-	-	-	cat	Ragdoll		female	no	negative	
9.	Kaposvár	+	+	-	-	-	cat	Ragdoll		female	no	negative	
10.	Kaposvár	+	+	-	-	-	cat	Ragdoll		female	no	negative	
11.	Kaposvár	+	+	-	-	-	cat	Ragdoll		female	no	negative	
12.	Kaposvár	+	-	-	-	-	cat	Ragdoll	+	female	no	negative	
13.	Kaposvár	+	-	-	-	-	cat	Ragdoll		female	no	negative	
14.	Kaposvár	+	-	-	-	-	cat	Ragdoll	+	male	no	negative	
LINK	Szántód	-	+	-	-	-	cat	European shorthair		-	no	negative	
1.	szthely/ Bársonytalp Z	+	-	-	-	-	cat	European shorthair		female	no	negative	
2.	Vörs	+	-	-	-	-	cat	European shorthair		female	no	negative	
3.	Keszthely	+	-	-	-	-	cat	European shorthair		female	no	negative	
4.	szthely/ Bársonytalp Z	+	-	-	-	-	cat	European shorthair		female	no	negative	
5.	szthely/ Barsonytalp Z	+	-	-	-	-	cat	European shorthair		female	no	negative	
6.	Balatonmogyoród	+	-	-	-	-	cat	European shorthair		male	no	negative	
7.	Rezi	+	-	-	-	-	cat	European shorthair		female	no	negative	
8.	Alsópáhok	+	-	-	-	-	cat	European shorthair		female	no	negative	
9.	?	+	-	-	-	-	cat	European shorthair		female	no	negative	
10.	Zalacsány	+	-	-	-	-	cat	European shorthair		male	yes	negative	
FJ1	Kecskemét	+	-	-	-	-	dog	French bulldog	+	-	yes	negative	
**FJ2**	**Kecskemét**	+	-	-	-	-	**cat**	**Ragdoll**	+	**female**	**yes**	**positive**	**100% *T. foetus***
**FJ3**	**Kecskemét**	+	-	-	-	-	**cat**	**Ragdoll**	+	**female**	**yes**	**positive**	**100% *T. foetus***
**FJ4**	**Kecskemét**	+	-	-	-	-	**dog**	**Maltese**	+	**male**	**yes**	**positive**	**100% *T. foetus***
**FJ5**	**Kecskemét**	+	-	-	-	-	**dog**			**-**	**yes**	**positive**	**100% *T. foetus***
**FJ6**	**Kecskemét**	+	-	-	-	-	**dog**			**-**	**yes**	**positive**	**100% *T. foetus***
**FJ8**	**Kecskemét**	+	-	-	-	-	**dog**			**-**	**yes**	**positive**	**100% *T. foetus***
**FJ9**	**Kecskemét**	+	-	-	-	-	**cat**	**Ragdoll**	+	**female**	**yes**	**positive**	**100% *T. foetus***
**FJ10**	**Kecskemét**	+	-	-	-	-	**cat**	**Ragdoll**	+	**male**	**yes**	**positive**	**100% *T. foetus***
YODA	Vecsés	+	-	-	-	-	cat	Maine coon		female	no	negative	
