# Supplementary material for: Molecular-phylogenetic investigation of trichomonads in dogs and cats reveals a novel Tritrichomonas species
Source: Parasit Vectors. 2024 Jun 26;17:271. doi: 10.1186/s13071-024-06343-0 (PMC11210186; doi:10.1186/s13071-024-06343-0)
Supplement: Supplementary file 3 — Supplementary material 3 Table S2. Distribution of PCR-positive and sequenced samples along with the GenBank accession numbers. [file 13071_2024_6343_MOESM3_ESM.docx]

|  | **PCR positive and sequenced** | | | **GenBank accession numbers** | | |
| --- | --- | --- | --- | --- | --- | --- |
|  | **18S rRNA gene: short** | **18S rRNA gene: long** | **ITS** | **18S rRNA gene: short** | **18S rRNA gene: long** | **ITS** |
| ***Tritrichomonas foetus*** | 17 | 6 | 17 | PP227422, PP227423 | PP227421 | PP239334, PP239335 |
| ***Pentatrichomonas hominis*** | 2 | 0 | 2 | PP227425 | 0 | PP239337 |
| ***Tritrichomonas* sp.** | 1 | 0 | 1 | PP227424 | 0 | PP239336 |
